# Temporal pattern in segmental motions of the foot in healthy senile adults: comparison between young and senile healthy adults

**DOI:** 10.1186/1757-1146-7-S1-A76

**Published:** 2014-04-08

**Authors:** Sang Gyo Seo, Dong Yeon Lee, Ji-Beom Kim, Seong Hyun Kim, Hye Sun Park, Hyo Jeong Yoo, Sung Ju Kim, Jihyeung Kim, Kyoung Min Lee, Chin Youb Chung, In Ho Choi

**Affiliations:** 1 Department of Orthopedic Surgery, Seoul National University Hospital, Seoul, Korea; 2 Department of statistics, Korea University, Seoul, Korea; 3 Department of Orthopedic Surgery, Seoul National University Boramae Medical Center, Seoul, Korea; 4 Department of Orthopedic Surgery, Seoul National University Bundang Hospital, Seongnam, Korea

## 

The incidence of foot and ankle disease increases as the age increases [1,2]. However, there was no report about differences of foot motion between senile person and young adults. The purpose of this study is to analyze distinctions according to age in segmental foot motion using 3D multi-foot model from healthy senior and young adults.

One hundred senile (50 males, 50 females) and young adults (50 males, 50 females) were tested by 3D multi-foot model with 15-markers. The cadence, speed, stride length, step width, step time, and stance phase were analyzed. The maximum and minimal values and motions of 3-planes of hallux, forefoot, hindfoot, and arch were compared between senile and young adults.

**Figure 1:**
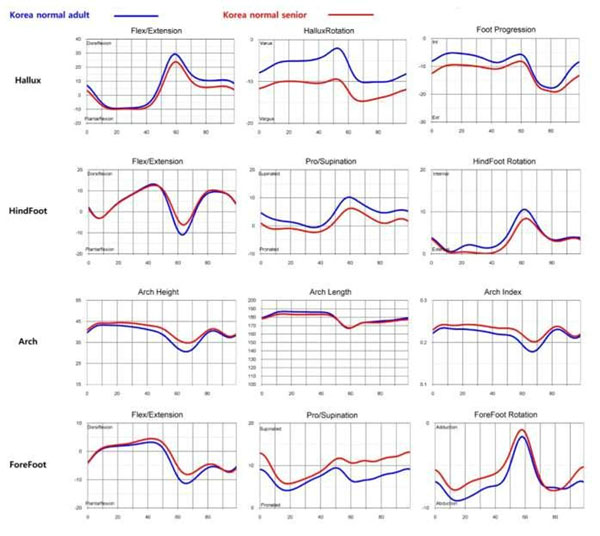
Comparison of the mean foot segmental motion between senile and young adults in female.

**Figure 2:**
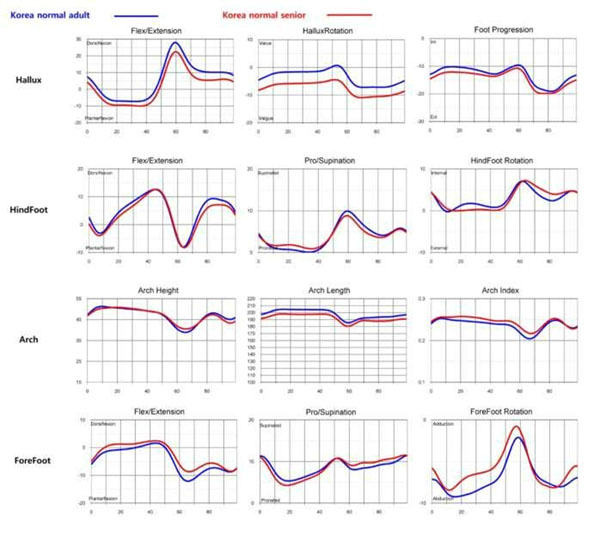
Comparison of the mean foot segmental motion between senile and young adults in male.

**Figure 3:**
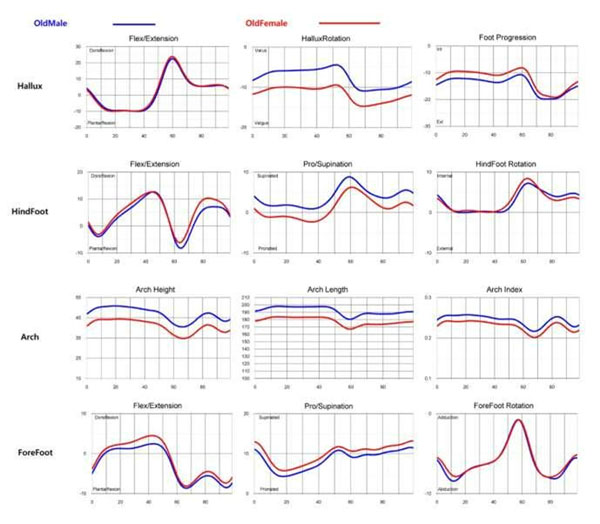
Comparison of the mean foot segmental motion between male and female in senior.

The cadence, speed, stride length, and step width were lower in senior. The stance phase was longer (Table1). In female, sagittal motion of all segment were more limited and hindfoot was more unstable in senior (Figure 1). In male, sagittal motion of hallux and forefoot were lower in senior (Figure 2). Hallux valgus of male and female was more severe in senior during gait. Arch height was no difference (Figure 3). In 3D foot gait analysis, the differences between senior and young adults were apparent. In summary, foot motion in senior had limited range of motion during gait. And hallux valgus in senior was more severe. But arch height was not diminished. The understanding about changes of foot segmental motion depending on age will suggest more correct approach in degenerative foot and ankle disease.

**Table 1:** Basic gait parameters in senile adults

	Male (mean ± SD)	Female (mean ± SD)	p- value
Cadence (cm)	109.3 ± 6.6	114.6 ± 6.9	< 0.001
Speed (cm/sec)	114.0 ± 9.2	111.5 ± 7.9	0.147
Stride length (cm)	124.5 ± 7.3	116.3 ± 7.4	< 0.001
Step width (cm)	62.4 ± 4.5	58.3 ± 4.1	< 0.001
Step time (sec)	0.55 ± 0.04	0.53 ± 0.03	< 0.001
Proportion of stance phase (%)	61.1 ± 1.1	60.6 ± 1.1	0.046
